# Supplementary material for: Performance, workload, and usability in a multiscreen, multi-device, information-rich environment
Source: PeerJ Comput Sci. 2018 Sep 10;4:e162. doi: 10.7717/peerj-cs.162 (PMC7924668; doi:10.7717/peerj-cs.162)
Supplement: Supplemental Information 1 [file peerj-cs-04-162-s001.docx]

**Statistical Analysis using Minitab (v18)**

**Dependent variable: Time (sec)**

Repeated-measures, single-factor ANOVA

GLM: Time (sec) versus Participant, Condition

Analysis of Variance

| Source | DF | Adj SS | Adj MS | F-Value | P-Value |
| --- | --- | --- | --- | --- | --- |
| Participant | 17 | 1440167 | 84716 | 6.53 | 0.000 |
| Cond | 2 | 11160 | 5580 | 0.43 | **0.654** |
| Error | 34 | 441094 | 12973 |  |  |
| Total | 53 | 1892421 |  |  |  |

**Dependent variable: Accuracy (number of errors)**

Friedman Test: Errors versus Cond blocked by Participant

S = 6.78 DF = 2 **P = 0.034**

S = 7.36 DF = 2 P = 0.025 (adjusted for ties)

Est Sum of

Cond N Median Ranks

A 16 6.333 30.5

B 16 5.667 25.5

C 16 7.500 40.0

Grand median = 6.500

Wilcoxon Signed Rank: A_Errors, B_Errors

Paired test for A_Errors - B_Errors

Test of median = 0.000000 versus median ≠ 0.000000

N for Wilcoxon Estimated

N N* Test Statistic P Median

Difference 16 2 13 64.0 0.208 1.000

Confidence

Estimated Achieved Interval

N N* Median Confidence Lower Upper

Difference 16 2 1.00 94.8 -0.50 2.00

Wilcoxon Signed Rank: A_Errors, C_Errors

Paired test for A_Errors - C_Errors

Test of median = 0.000000 versus median ≠ 0.000000

N for Wilcoxon Estimated

N Test Statistic P Median

Difference 18 18 53.5 0.170 -1.000

Confidence

Estimated Achieved Interval

N Median Confidence Lower Upper

Difference 18 -1.00 95.0 -2.00 0.50

Wilcoxon Signed Rank: B_Errors, C_Errors

Paired test for B_Errors - C_Errors

Test of median = 0.000000 versus median ≠ 0.000000

N for Wilcoxon Estimated

N N* Test Statistic P Median

Difference 16 2 14 13.0 **0.014** -1.750

Confidence

Estimated Achieved Interval

N N* Median Confidence Lower Upper

Difference 16 2 -1.75 94.8 -3.00 -0.50

**Dependent variable: Workload (NASA TLX scores)**

NASA TLX Subscales: Mental Demand (MD), Physical Demand (PD), Temporal Demand (TD), Performance (Perf), Effort, Frustration (Frust), and Total Composite TLX score (Tot_Comp).

Friedman Test: MD versus COND blocked by SUBJ

S = 1.08 DF = 2 P = 0.582

S = 1.26 DF = 2 P = 0.533 (adjusted for ties)

Sum of

COND N Est Median Ranks

A 18 53.333 39.5

B 18 46.667 33.5

C 18 47.500 35.0

Grand median = 49.167

Friedman Test: PD versus COND blocked by SUBJ

S = 0.58 DF = 2 P = 0.747

S = 0.81 DF = 2 P = 0.668 (adjusted for ties)

Sum of

COND N Est Median Ranks

A 18 17.500 35.5

B 18 17.500 34.0

C 18 17.500 38.5

Grand median = 17.500

Friedman Test: TD versus COND blocked by SUBJ

S = 1.19 DF = 2 P = 0.550

S = 1.41 DF = 2 P = 0.494 (adjusted for ties)

Sum of

COND N Est Median Ranks

A 18 39.167 36.5

B 18 35.833 32.5

C 18 40.000 39.0

Grand median = 38.333

Friedman Test: PERF versus COND blocked by SUBJ

S = 1.36 DF = 2 P = 0.506

S = 1.63 DF = 2 P = 0.442 (adjusted for ties)

Sum of

COND N Est Median Ranks

A 18 32.500 38.5

B 18 32.500 37.5

C 18 30.000 32.0

Grand median = 31.667

Friedman Test: EFFORT versus COND blocked by SUBJ

S = 1.19 DF = 2 P = 0.550

S = 1.46 DF = 2 P = 0.482 (adjusted for ties)

Sum of

COND N Est Median Ranks

A 18 44.167 39.0

B 18 38.333 32.5

C 18 42.500 36.5

Grand median = 41.667

Friedman Test: FRUST versus COND blocked by SUBJ

S = 0.78 DF = 2 P = 0.678

S = 0.93 DF = 2 P = 0.627 (adjusted for ties)

Sum of

COND N Est Median Ranks

A 18 25.000 38.0

B 18 23.333 37.0

C 18 21.667 33.0

Grand median = 23.333

Friedman Test: TOT_COMP versus COND blocked by SUBJ

S = 2.19 DF = 2 P = 0.334

S = 2.43 DF = 2 P = 0.297 (adjusted for ties)

Sum of

COND N Est Median Ranks

A 18 40.208 39.5

B 18 37.708 31.0

C 18 39.792 37.5

Grand median = 39.236

**Dependent variable: Usability (CSUQ scores)**

CSUQ Subscales: Overall satisfaction, System usefulness, Information quality, Interface quality.

Repeated-measures, single-factor ANOVA

GLM: System Usefulness versus Subject, Condition

Analysis of Variance

| Source | DF | Adj SS | Adj MS | F-Value | P-Value |
| --- | --- | --- | --- | --- | --- |
| SUBJ | 17 | 26.701 | 1.5706 | 1.83 | 0.066 |
| COND | 2 | 7.338 | 3.6690 | 4.27 | **0.022** |
| Error | 34 | 29.202 | 0.8589 |  |  |
| Total | 53 | 63.241 |  |  |  |

Repeated-measures, single-factor ANOVA

GLM: Information Quality versus Subject, Condition

Analysis of Variance

| Source | DF | Adj SS | Adj MS | F-Value | P-Value |
| --- | --- | --- | --- | --- | --- |
| SUBJ | 17 | 38.330 | 2.2547 | 7.54 | 0.000 |
| COND | 2 | 2.260 | 1.1301 | 3.78 | **0.033** |
| Error | 34 | 10.166 | 0.2990 |  |  |
| Total | 53 | 50.756 |  |  |  |

Friedman Test: OVERALL versus COND blocked by SUBJ

S = 12.19 DF = 2 **P = 0.002**

S = 12.37 DF = 2 P = 0.002 (adjusted for ties)

Sum of

COND N Est Median Ranks

A 18 5.0509 27.0

B 18 6.0046 47.5

C 18 5.4861 33.5

Grand median = 5.5139

Friedman Test: INTER QUAL versus COND blocked by SUBJ

S = 14.53 DF = 2 **P = 0.001**

S = 16.87 DF = 2 P = 0.000 (adjusted for ties)

Sum of

COND N Est Median Ranks

A 18 5.1111 27.5

B 18 6.0556 49.0

C 18 5.3333 31.5

Grand median = 5.5000

Paired T-Test and CI: A-Sys Use, B-Sys Use

Descriptive Statistics

| Sample | N | Mean | StDev | SE Mean |
| --- | --- | --- | --- | --- |
| A-Sys Use | 18 | 5.000 | 1.062 | 0.250 |
| B-Sys Use | 18 | 5.869 | 1.034 | 0.244 |

Estimation for Paired Difference

| Mean | StDev | SE Mean | 95% CI for μ_difference |
| --- | --- | --- | --- |
| -0.869 | 1.467 | 0.346 | (-1.598, -0.140) |

*µ_difference: mean of (A-Sys Use - B-Sys Use)*

Test

| Null hypothesis | H₀: μ_difference = 0 |
| --- | --- |
| Alternative hypothesis | H₁: μ_difference ≠ 0 |

| T-Value | P-Value |
| --- | --- |
| -2.51 | **0.022** |

Paired T-Test and CI: A-Sys Use, C-Sys Use

Descriptive Statistics

| Sample | N | Mean | StDev | SE Mean |
| --- | --- | --- | --- | --- |
| A-Sys Use | 18 | 5.000 | 1.062 | 0.250 |
| C-Sys Use | 18 | 5.222 | 1.045 | 0.246 |

Estimation for Paired Difference

| Mean | StDev | SE Mean | 95% CI for μ_difference |
| --- | --- | --- | --- |
| -0.222 | 1.487 | 0.350 | (-0.962, 0.517) |

*µ_difference: mean of (A-Sys Use - C-Sys Use)*

Test

| Null hypothesis | H₀: μ_difference = 0 |
| --- | --- |
| Alternative hypothesis | H₁: μ_difference ≠ 0 |

| T-Value | P-Value |
| --- | --- |
| -0.63 | 0.534 |

Paired T-Test and CI: B-Sys Use, C-Sys Use

Descriptive Statistics

| Sample | N | Mean | StDev | SE Mean |
| --- | --- | --- | --- | --- |
| B-Sys Use | 18 | 5.869 | 1.034 | 0.244 |
| C-Sys Use | 18 | 5.222 | 1.045 | 0.246 |

Estimation for Paired Difference

| Mean | StDev | SE Mean | 95% CI for μ_difference |
| --- | --- | --- | --- |
| 0.647 | 0.889 | 0.210 | (0.205, 1.089) |

*µ_difference: mean of (B-Sys Use - C-Sys Use)*

Test

| Null hypothesis | H₀: μ_difference = 0 |
| --- | --- |
| Alternative hypothesis | H₁: μ_difference ≠ 0 |

| T-Value | P-Value |
| --- | --- |
| 3.09 | **0.007** |

Paired T-Test and CI: A-Info Qual, B-Info Qual

Descriptive Statistics

| Sample | N | Mean | StDev | SE Mean |
| --- | --- | --- | --- | --- |
| A-Info Qual | 18 | 5.130 | 1.006 | 0.237 |
| B-Info Qual | 18 | 5.620 | 0.970 | 0.229 |

Estimation for Paired Difference

| Mean | StDev | SE Mean | 95% CI for μ_difference |
| --- | --- | --- | --- |
| -0.491 | 0.807 | 0.190 | (-0.892, -0.089) |

*µ_difference: mean of (A-Info Qual - B-Info Qual)*

Test

| Null hypothesis | H₀: μ_difference = 0 |
| --- | --- |
| Alternative hypothesis | H₁: μ_difference ≠ 0 |

| T-Value | P-Value |
| --- | --- |
| -2.58 | **0.020** |

Paired T-Test and CI: A-Info Qual, C-Info Qual

Descriptive Statistics

| Sample | N | Mean | StDev | SE Mean |
| --- | --- | --- | --- | --- |
| A-Info Qual | 18 | 5.130 | 1.006 | 0.237 |
| C-Info Qual | 18 | 5.463 | 0.949 | 0.224 |

Estimation for Paired Difference

| Mean | StDev | SE Mean | 95% CI for μ_difference |
| --- | --- | --- | --- |
| -0.333 | 0.907 | 0.214 | (-0.785, 0.118) |

*µ_difference: mean of (A-Info Qual - C-Info Qual)*

Test

| Null hypothesis | H₀: μ_difference = 0 |
| --- | --- |
| Alternative hypothesis | H₁: μ_difference ≠ 0 |

| T-Value | P-Value |
| --- | --- |
| -1.56 | 0.138 |

Paired T-Test and CI: B-Info Qual, C-Info Qual

Descriptive Statistics

| Sample | N | Mean | StDev | SE Mean |
| --- | --- | --- | --- | --- |
| B-Info Qual | 18 | 5.620 | 0.970 | 0.229 |
| C-Info Qual | 18 | 5.463 | 0.949 | 0.224 |

Estimation for Paired Difference

| Mean | StDev | SE Mean | 95% CI for μ_difference |
| --- | --- | --- | --- |
| 0.157 | 0.564 | 0.133 | (-0.123, 0.438) |

*µ_difference: mean of (B-Info Qual - C-Info Qual)*

Test

| Null hypothesis | H₀: μ_difference = 0 |
| --- | --- |
| Alternative hypothesis | H₁: μ_difference ≠ 0 |

| T-Value | P-Value |
| --- | --- |
| 1.18 | 0.253 |

Wilcoxon Signed Rank: A-Overall, B-Overall

Data Display (WRITE)

Data

| Paired test for A-Overall - B-Overall |
| --- |

Wilcoxon Signed Rank Test: Difference

Method

| η: median of Difference |
| --- |

Descriptive Statistics

| Sample | N | Median |
| --- | --- | --- |
| Difference | 18 | -0.972222 |

Test

| Null hypothesis | H₀: η = 0 |
| --- | --- |
| Alternative hypothesis | H₁: η ≠ 0 |

| Sample | N for Test | Wilcoxon Statistic | P-Value |
| --- | --- | --- | --- |
| Difference | 18 | 29.50 | **0.016** |

Wilcoxon Signed Rank CI: Difference

Method

Wilcoxon Signed Rank: A-Overall, C-Overall

Data

| Paired test for A-Overall - C-Overall |
| --- |

Method

| η: median of Difference |
| --- |

Descriptive Statistics

| Sample | N | Median |
| --- | --- | --- |
| Difference | 18 | -0.305556 |

Test

| Null hypothesis | H₀: η = 0 |
| --- | --- |
| Alternative hypothesis | H₁: η ≠ 0 |

| Sample | N for Test | Wilcoxon Statistic | P-Value |
| --- | --- | --- | --- |
| Difference | 18 | 50.00 | 0.127 |

Wilcoxon Signed Rank: B-Overall, C-Overall

Data

| Paired test for B-Overall - C-Overall |
| --- |

Method

| η: median of Difference |
| --- |

Descriptive Statistics

| Sample | N | Median |
| --- | --- | --- |
| Difference | 18 | 0.5 |

Test

| Null hypothesis | H₀: η = 0 |
| --- | --- |
| Alternative hypothesis | H₁: η ≠ 0 |

| Sample | N for Test | Wilcoxon Statistic | P-Value |
| --- | --- | --- | --- |
| Difference | 17 | 132.50 | **0.009** |

Wilcoxon Signed Rank: A-Inter Qual, B-Inter Qual

Data

| Paired test for A-Inter Qual - B-Inter Qual |
| --- |

Method

| η: median of Difference |
| --- |

Descriptive Statistics

| Sample | N | Median |
| --- | --- | --- |
| Difference | 18 | -1 |

Test

| Null hypothesis | H₀: η = 0 |
| --- | --- |
| Alternative hypothesis | H₁: η ≠ 0 |

| Sample | N for Test | Wilcoxon Statistic | P-Value |
| --- | --- | --- | --- |
| Difference | 16 | 14.00 | **0.006** |

Wilcoxon Signed Rank: A-Inter Qual, C-Inter Qual

Data

| Paired test for A-Inter Qual - C-Inter Qual |
| --- |

Method

| η: median of Difference |
| --- |

Descriptive Statistics

| Sample | N | Median |
| --- | --- | --- |
| Difference | 18 | -0.333333 |

Test

| Null hypothesis | H₀: η = 0 |
| --- | --- |
| Alternative hypothesis | H₁: η ≠ 0 |

| Sample | N for Test | Wilcoxon Statistic | P-Value |
| --- | --- | --- | --- |
| Difference | 15 | 46.00 | 0.443 |

Wilcoxon Signed Rank: B-Inter Qual, C-Inter Qual

Data

| Paired test for B-Inter Qual - C-Inter Qual |
| --- |

Method

| η: median of Difference |
| --- |

Descriptive Statistics

| Sample | N | Median |
| --- | --- | --- |
| Difference | 18 | 0.666667 |

Test

| Null hypothesis | H₀: η = 0 |
| --- | --- |
| Alternative hypothesis | H₁: η ≠ 0 |

| Sample | N for Test | Wilcoxon Statistic | P-Value |
| --- | --- | --- | --- |
| Difference | 14 | 95.00 | **0.008** |
