# Supplementary material for: Performance, workload, and usability in a multiscreen, multi-device, information-rich environment
Source: PeerJ Comput Sci. 2018 Sep 10;4:e162. doi: 10.7717/peerj-cs.162 (PMC7924668; doi:10.7717/peerj-cs.162)
Supplement: Supplemental Information 6 [file peerj-cs-04-162-s006.docx]

**Debrief Interview Questions**

1. In your opinion, did any of the three layouts stand out as the easiest to use when utilizing all the different forms of information needed to complete the tasks? If so, which layout and why?
2. For the multiscreen and multi-device conditions, did you find the partitioning of information and functions across the different screens helpful for focusing your attention or distracting? Please explain.
3. Could you anticipate using one of these layouts on a daily basis to complete course work, projects, professional tasks, or other tasks? If so, which layout and why?
4. Did the organization of information used for layout B (dual monitor, single tablet) optimize your performance? Why or why not?
5. Did the organization of information used for layout C (single monitor, dual tablet) optimize your performance? Why or why not?
6. If you were given the same tasks as this project and given layout B, how would you have organized the information needed to complete the task?
7. If you had the ability to create your own work area computing arrangement for complex tasks, what would it include (number and size of monitors, number of mobile devices, types of devices and sizes)?
   1. How would you distribute the information and functions from the scenarios you completed across each screen and device?
8. Did your background with computers or tablets make completing these tasks easier or more difficult?
9. If your future employer provided you with a dual monitor workstation how would you utilize the dual screens? Would you make one screen a “home” screen and the other a “supporting” screen? How would you decide which screen is which?
10. Is there anything else you’d like to comment on with respect to this experiment?
