# Supplementary material for: Performance, workload, and usability in a multiscreen, multi-device, information-rich environment
Source: PeerJ Comput Sci. 2018 Sep 10;4:e162. doi: 10.7717/peerj-cs.162 (PMC7924668; doi:10.7717/peerj-cs.162)
Supplement: Supplemental Information 7 [file peerj-cs-04-162-s007.doc]

# CSUQ: Computer System Usability Questionnaire

# Please rate the usability of the system.

# strongly disagree strongly agree

| 1. | Overall, I am satisfied with how easy it is to use this system. | 1 | 2 | 3 | 4 | 5 | 6 | 7 |
| --- | --- | --- | --- | --- | --- | --- | --- | --- |
| 2. | It was simple to use this system. | 1 | 2 | 3 | 4 | 5 | 6 | 7 |
| 3. | I can effectively complete my work using this system. | 1 | 2 | 3 | 4 | 5 | 6 | 7 |
| 4. | I am able to complete my work quickly using this system. | 1 | 2 | 3 | 4 | 5 | 6 | 7 |
| 5. | I am able to efficiently complete my work using this system. | 1 | 2 | 3 | 4 | 5 | 6 | 7 |
| 6. | I feel comfortable using this system. | 1 | 2 | 3 | 4 | 5 | 6 | 7 |
| 7. | It was easy to learn to use this system. | 1 | 2 | 3 | 4 | 5 | 6 | 7 |
| 8. | I believe I became productive quickly using this system. | 1 | 2 | 3 | 4 | 5 | 6 | 7 |
| 9. | The system gives error messages that clearly tell me how to fix problems. | 1 | 2 | 3 | 4 | 5 | 6 | 7 |
| 10. | Whenever I make a mistake using the system, I recover easily and quickly. | 1 | 2 | 3 | 4 | 5 | 6 | 7 |
| 11. | The information (such as online help, on-screen messages, and other documentation) provided with this system is clear. | 1 | 2 | 3 | 4 | 5 | 6 | 7 |
| 12. | It is easy to find the information I needed. | 1 | 2 | 3 | 4 | 5 | 6 | 7 |
| 13. | The information provided for the system is easy to understand. | 1 | 2 | 3 | 4 | 5 | 6 | 7 |
| 14. | The information is effective in helping me complete the tasks and scenarios. | 1 | 2 | 3 | 4 | 5 | 6 | 7 |
| 15. | The organization of the information on the systems screens is clear. | 1 | 2 | 3 | 4 | 5 | 6 | 7 |
| 16. | The interface of the system is pleasant. | 1 | 2 | 3 | 4 | 5 | 6 | 7 |
| 17. | I like using the interface of this system. | 1 | 2 | 3 | 4 | 5 | 6 | 7 |
| 18. | This system has all the functions and capabilities I expect it to have. | 1 | 2 | 3 | 4 | 5 | 6 | 7 |
| 19. | Overall, I am satisfied with this system. | 1 | 2 | 3 | 4 | 5 | 6 | 7 |

List the most **negative** aspects

1.

2.

3.

List the most **positive** aspects.

1.

2.

3.
